# Collagen Proportionate Area and Histological Grading of Fibrosis as Predictors of Clinical Outcomes and Mortality in Patients with Steatotic Liver Disease

**DOI:** 10.1016/j.jceh.2025.103464

**Published:** 2026-01-02

**Authors:** Andreas Bartholdy, Pernille Y. Nielsen, Lise L. Gluud, Laust H. Mortensen, Birgitte M. Viuff, Elisabeth D. Galsgaard, Majken K. Jensen

**Affiliations:** ∗ Department of Public Health, University of Copenhagen, Copenhagen, Denmark; † Department of Applied Mathematics and Computer Science, Technical University of Denmark, 2800, Kongens Lyngby, Denmark; ‡ Department of Clinical Medicine, Faculty of Health and Medical Sciences, University of Copenhagen, Copenhagen, Denmark; § Gastro Unit, Copenhagen University Hospital Hvidovre, Hvidovre 2650, Denmark; ¦ Research and Early Development, Novo Nordisk A/S, Måløv, Denmark

**Keywords:** liver fibrosis, collagen, steatotic liver disease, steatohepatitis

## Abstract

**Background/Aims:**

Steatotic liver disease (SLD) including metabolic dysfunction-associated and alcoholic liver disease, is the most prevalent chronic liver condition globally. Liver biopsy remains the gold standard for fibrosis assessment, but traditional staging is difficult even for highly skilled experts. Collagen proportionate area (CPA), a quantitative digital pathology measure, may offer an alternative, yet its prognostic value in population-based settings is unclear.

**Methods:**

Liver biopsies from a cohort of 166 adults with biopsy-confirmed SLD, diagnosed between 1995 and 2008, were retrieved. The liver biopsies underwent fibrosis staging (stages F0–F4) by a pathologist and digital CPA quantification. Clinical outcomes and mortality were tracked via national registries over a median follow-up of 14.8 years. Associations between fibrosis stage, CPA, and all-cause mortality and liver-related events were assessed using Cox regression and cause-specific hazard models, adjusting for alcohol use and age. Predictive performance was evaluated with area under the receiver operating characteristic curve (AUC), index of prediction accuracy (IPA), and calibration plots, internally validated by bootstrapping.

**Results:**

CPA correlated significantly with fibrosis stage (Spearman's ρ = 0.63), particularly in advanced fibrosis. Higher fibrosis stages and greater CPA were associated with increased all-cause mortality and liver-related events, but the associations for CPA were nonsignificant after adjustment for age and alcohol use. Scaled estimates of HRs from CPA were lower compared to HRs from fibrosis stages. Predictive models for mortality demonstrated comparable and moderate discrimination for CPA and fibrosis stage, improving with age adjustment. IPA values and calibration plots indicated positive predictive accuracy for mortality but poor performance for liver-specific outcomes.

**Conclusions:**

CPA closely correlates with fibrosis stage and has comparable long-term prognostic value in SLD. Interpretation of study results is limited by the small sample size and low number of liver-specific events. Larger studies are needed to validate CPA's clinical utility and explore its integration into routine practice.

## Graphical abstract

**Figure:**
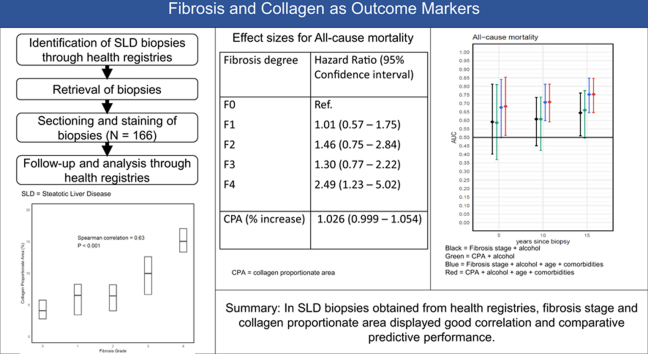

Steatotic liver disease (SLD), encompassing metabolic dysfunction-associated liver disease (MASLD), alcoholic liver disease (ALD), and a combination of the two (MetALD), has emerged as the most prevalent chronic liver disease globally, affecting an estimated one-third of the adult population.^1^^,^^2^ It is closely linked to the rising incidence of obesity, type 2 diabetes, and metabolic syndrome, as well as increases in alcohol consumption,^3^, ^4^, ^5^ SLD encompasses a spectrum of liver pathology ranging from simple steatosis to steatohepatitis, progressive fibrosis, cirrhosis, and hepatocellular carcinoma.^6^ As a leading cause of liver-related morbidity and mortality, MASLD in particular is projected to become the most common indication for liver transplantation in many countries.^7^

Despite recent advances in noninvasive fibrosis assessment tests, liver biopsy remains the gold standard for assessing both the presence of steatohepatitis and accurate fibrosis staging.^6^^,^^8^^,^^9^ However, traditional histopathological evaluation is labor-intensive and prone to interobserver variability, particularly in the assessment of features such as ballooning degeneration and fibrosis stage.^10^, ^11^, ^12^ These limitations pose challenges for both clinical decision-making and the evaluation of treatment efficacy in clinical trials. Moreover, fibrosis staging with categorical progression assessment may not fully capture the biological continuum of disease progression or provide sufficient granularity for risk stratification.

In recent years, digital pathology and automated image analysis have emerged as promising tools to enhance the objectivity, reproducibility, and scalability of liver biopsy evaluation.^13^ Among these, collagen proportionate area (CPA) has shown potential as a more sensitive and quantitative alternative to traditional fibrosis staging.^14^, ^15^, ^16^ However, real-world data from registry-based settings on SLD disease remain limited, and no prior studies have integrated CPA with long-term outcomes using registry-based mortality data. Health data registries such as those in Denmark, provide a unique source for obtaining historical, real-world data. Furthermore, formalin-fixed, paraffin-embedded tissue specimens are mostly kept indefinitely in Danish pathology departments and can be requisitioned for further diagnostics and research purposes.^17^

By linking histological data with national health registries, we created a registry-linked cohort of patients with biopsy-confirmed SLD and extensive long-term follow-up. This dataset enables us to follow SLD patients and compare the prognostic value of CPA with fibrosis staging. To our knowledge, this is the first registry-linked study to include both MASLD and ALD, combining digital histological quantification with long-term, registry-based follow-up and proper internal validation of findings. The primary aims of this study were to examine the correlation between CPA and fibrosis stage, evaluate their associations with long-term clinical outcomes, and assess the comparative prognostic performance of these two histological measures.

## MATERIALS AND METHODS

### Population

The cohort was defined using Danish national registries, accessed through Statistics Denmark. The registries used were the Danish National Patient Registry, the National Registry for Pathology (NRP), the Prescription Registry, and the Cause of Death Registry.^18^, ^19^, ^20^, ^21^ Coupling of information from different registries was made possible by the Danish Civil Registration System.^22^ The coupling and characteristics of patients in NRP have been described in previous work.^23^

**Figure 1:**
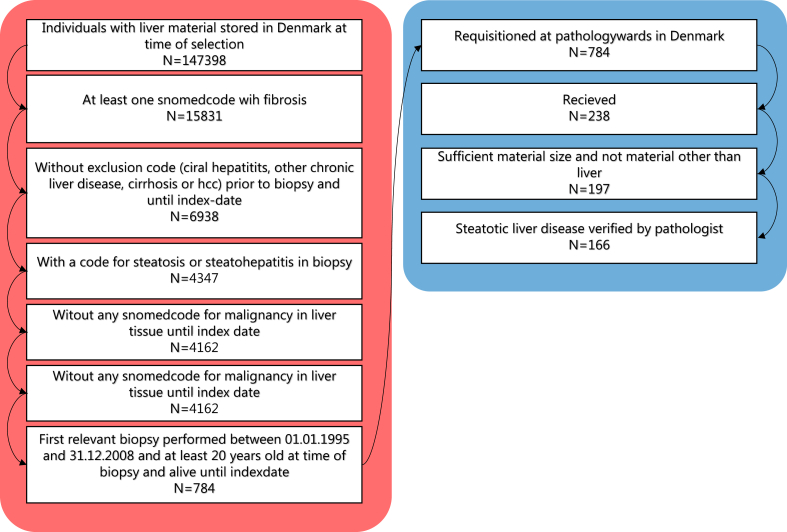
Flowchart of cohort selection. Red part (left) represents selection steps prior to obtaining biopsies. Blue part (right) represents biopsy obtainment and postprocessing selection.

The overall selection and exclusion steps are summarized in Figure 1. To identify the cohort, we first selected all patients over the age of 18 with a first-time liver biopsy (SNOMED: T56) showing fibrosis and a diagnosis of steatosis between January 1, 1995 and December 31, 2008 (see Supplementary 1). We then excluded patients with liver diseases other than SLD prior to or up to 180 days after the index biopsy. This identified 784 individuals with biopsy-verified steatotic liver disease (SLD).

The formalin-fixed, paraffin-embedded biopsies were then requested from Danish pathology departments, where biopsies are stored. Of the 784 individuals, we were able to obtain the original biopsies from 238 individuals. After biopsy processing, 72 individuals were removed, either due to insufficient biopsy material for diagnosis, only healthy liver tissue in the biopsy, or a diagnosis other than SLD based on the pathology assessment of the retrieved biopsy. The remaining 166 patients were included in the study.

### Histopathological Assessment

Received specimens were screened at the Pathology Department, Copenhagen University Hospital, Hvidovre. Specimens with tissue other than liver or those with insufficient material were removed. The remaining liver specimens were sectioned and subjected to a panel of histological and immunohistochemical stains. Upon pathologist evaluation, liver specimens with other liver pathology or with no pathology were removed. For details and specific codes, see Supplement 1.

Staining included hematoxylin-eosin and Picrosirius red. The stained slides were digitized using a high-resolution whole-slide scanner (e.g. Hamamatsu NanoZoomer or equivalent). Digital images were analyzed using the Visiopharm® image analysis software (Hørsholm, Denmark), which enabled automated segmentation and quantification of positively stained areas. Following automatic biopsy detection, each image was manually reviewed and edited to exclude artifacts such as folds, droplets, and tissue edges. CPA was calculated as the percentage of positive staining relative to the total tissue area.

In addition to automated analysis, all biopsy samples were independently reviewed by an experienced liver pathologist not otherwise involved in this study who was blinded to clinical data. Fibrosis staging and histological grading were performed according to the non-alcoholic steatohepatitis Clinical Research Network (NASH-CRN) criteria. Fibrosis was staged on a scale from F0 to F4.^24^ Steatosis, lobular inflammation, and hepatocellular ballooning were also assessed and graded, and a composite non-alcoholic fatty liver disease (NAFLD) Activity Score (NAS) was calculated.^24^^,^^25^

### Outcomes and Variables

Codes used for the outcome definitions and covariates are provided in Supplementary 2–3. Patients were followed from 180 days after the index biopsy until death, emigration, or end of follow-up (December 31st, 2023). The primary outcome was all-cause mortality. We also defined a secondary compound outcome of liver events, consisting of decompensated liver events liver cancer, esophageal varices without bleeding, and liver-related death. Because we only had data on clinical diagnoses until the end of 2021, but information on death dates updated through 2023, different end dates were used in the respective outcome analyses. Other baseline characteristics were age at diagnosis, sex, type 2 diabetes, hypertension, dyslipidemia, and excessive alcohol consumption. Alcohol consumption is not registered within the Danish registries. Instead, we estimated excessive alcohol consumption defined as any primary or secondary diagnostic code for excessive alcohol consumption or for diseases with an alcoholic etiology prior to or up to 180 days after initial biopsy. Prescriptions were used to assess type 2 diabetes, and dyslipidemia. Two purchases within a timeframe of 180 days were used to define these conditions, with type 2 diabetes further requiring at least one purchase of a noninsulin antidiabetic in the same period.

### Statistics

Baseline characteristics were summarized using descriptive statistics, with continuous variables presented as medians with interquartile ranges (IQR) and categorical variables reported as counts and percentages. Frequency data were aggregated to ensure no reported counts were below 3. Correlation between fibrosis stage and CPA values was assessed using Spearman's rank correlation coefficient.

Cox proportional hazards regression models were used to evaluate associations between fibrosis stage and all-cause mortality, as well as between CPA values and all-cause mortality. We calculated both crude models, including only alcohol overconsumption, and adjusted models. The adjusted models included alcohol, age, hypertension, and type 2 diabetes because these variables may influence the likelihood of being diagnosed at a particular fibrosis stage and mortality. For associations with liver-related outcomes, cause-specific hazards regression was employed, treating death from non–liver-related causes as a competing event. Patients were censored at emigration or the end of follow-up (December 31, 2021). The proportional hazards assumption was assessed using Schoenfeld residuals, and the linearity of continuous predictors was assessed via martingale residuals. Because fibrosis stage is a categorical variable and CPA is continuous, direct comparison of their hazard ratios (HRs) is not straightforward. To ease comparison, we also calculated scaled estimates based on differences in median CPA values between fibrosis groups and individuals without fibrosis (F0). The scaled estimates are presented alongside the original per-unit increase HRs from the models.

To investigate a potential interaction between alcohol overconsumption and all-cause mortality, or whether alcohol overconsumption could be included in the model as a regular covariate, Cox models with and without an interaction term were compared. Model fit was assessed using the Akaike information criterion (AIC), and differences were tested via likelihood ratio tests.

Predictive performance of fibrosis stage and CPA values was evaluated by calculating the area under the receiver operating characteristic curve (AUC), index of prediction accuracy (IPA),^26^ and by generating calibration plots for Cox and cause-specific hazard models. As prediction performance for survival models is time-specific, all metrics were evaluated at 5, 10, and 15 years postbiopsy.

Internal validation to correct for overfitting was performed using 500 bootstrap resamples. Given that prior studies have not consistently applied internal validation, “naïve” estimates (i.e. performance metrics calculated by evaluating the models on the same dataset used for model fitting) are also reported in the supplementary materials for comparison. No external validation cohort was available.

## RESULTS

Baseline characteristics are presented in Table 1. The median age was 54 years, and approximately half were male (58%). Approximately one-third of the patients had prior registration consistent with alcohol overconsumption. Standardized mean differences between retrieved and nonretrieved biopsies were generally small, with the only noticeable difference observed for sex (Supplementary 9).

**Table 1:** Baseline Characteristics for Included Patients.

Variable	Value
N total	166
Age, years, median (Q1-Q3)	54 (45–61)
Male sex, n (%)	96 (58%)
Fibrosis stage	
F0, n (%)	44 (27%)
F1, n (%)	43 (26%)
F2, n (%)	23 (14%)
F3, n (%)	42 (25%)
F4, n (%)	14 (8%)
Steatohepatitis	109 (66%)
NAS score, median (Q1-Q3)	4 (3–5.75)
Steatosis	
1	39 (23%)
2	64 (39%)
3	63 (38%)
Inflammation	
0	57 (34 %)
1	44 (27%)
2	65 39 (%)
Ballooning	
0	57 (34%)
1	44 (27%)
2	65 (39%)
CPA, median (Q1-Q3)	6.6% (4.1–11.3)
Diabetes	18 (11%)
Hypertension	74 (45%)
Dyslipidemia	7 (4%)
Alcohol overconsumption	60 (36%)

CPA, collagen proportionate area; NAS, NAFLD activity score.

### Correlation Between Fibrosis Stage and CPA Values

**Figure 2:**
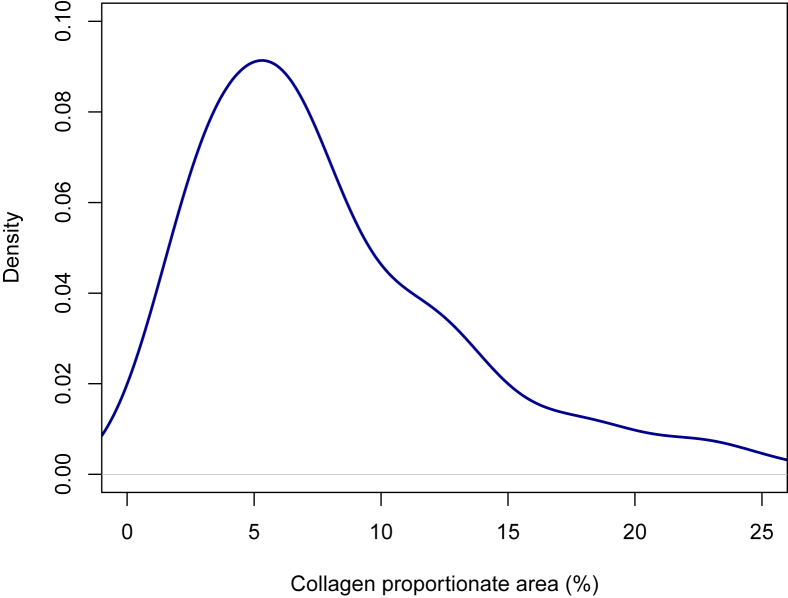
Density plot of the distribution of CPA values. Y-axis is density of observations. X-axis is truncated to prevent identification of outliers. CPA, collagen proportionate area.

**Figure 3:**
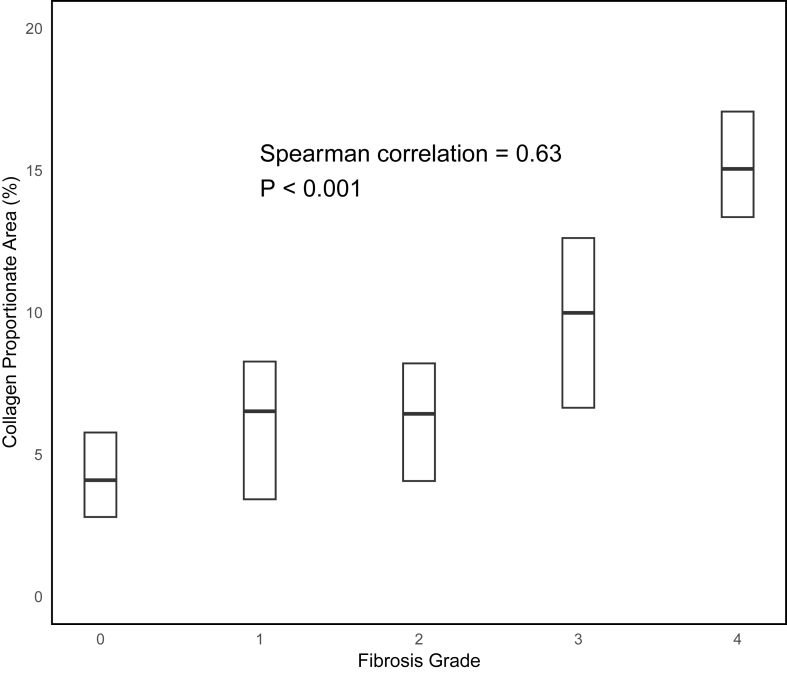
Distribution of CPA. Median values and quartiles by fibrosis stage. Whisker and outliers are removed to prevent identification of outliers. CPA, collagen proportionate area.

The distribution of CPA values is visualized in Figure 2. CPA values were somewhat skewed with a median of 6.6% and an interquartile range (IQR) of 4.1%–11.4%. The median and IQR of CPA values across fibrosis stages were 4.1% (2.8–5.8) in F0, 6.5% (3.4–8.3) in F1, 6.4% (4.1–8.2) in F2, 10.4% (6.8–13.2) in F3, and 19.4% (15.5–24.1) in F4. The correlation between fibrosis stage and CPA was significant, with a Spearman correlation of 0.63 (*P* < 0.001) (Figure 3). The correlation was nonlinear and appeared to be mostly driven by a high correlation among the higher fibrosis stages.

### Fibrosis, CPA, and Clinical Outcomes

Over a mean follow-up of 14.8 (IQR: 7.8–21.7) years, 112 out of 166 individuals died. In univariate regression, there was a monotonic relationship between fibrosis stage and HR for all-cause mortality when compared with individuals without fibrosis. Only those with F4 had a statistically significant increase in HR for all-cause mortality compared to F0, with an HR of 3.66 (95% CI: 1.85–7.25). When further adjusting for age at biopsy, sex, hypertension, and type 2 diabetes, results for F4 remained significant but were somewhat attenuated with an HR of 2.49 (95% CI: 1.23–5.02) (Table 2).

**Table 2:** Hazard Ratios (HR) for Fibrosis Assessed by Pathologists or by Collagen Proportionate Area (CPA) and All-Cause Mortality. Model 1: Unadjusted. Model 2: Adjusted for Alcohol. Model 3: Further Adjusted for Age, Hypertension, and Type 2 Diabetes Mellitus. Scaled CPA Estimates are Obtained by Applying the HR per % CPA Increase to the Difference in Mean CPA Values Between F1–F4 and F0.

	Variable	Model 1	Model 2	Model 3
		HR	HR	HR
Fibrosis stage	F0 F1 F2 F3 F4	Ref. 1.20 (0.70–2.07) 1.46 (0.78–2.80) 1.57 (0.91–2.60) 3.66 (1.85–7.25)	Ref. 1.23 (0.71–2.13) 1.59 (0.83–2.03) 1.54 (0.91–2.60) 3.54 (1.78–7.02)	Ref. 1.01 (0.57–1.75) 1.46 (0.75–2.84) 1.30 (0.77–2.22) 2.49 (1.23–5.02)
CPA Scaled CPA estimates	CPA (% increase) CPA 4.10 % (F0) CPA 6.52 % (F1) CPA 6.43 % (F2) CPA 10.42 % (F3) CPA 19.36 % (F4)	1.038 (1.014–1.066) Ref. 1.010 (1.03–1.17) 1.09 (1.03–1.15) 1.27 (1.15–1.41) 1.76 (1.38–2.22)	1.032 (1.004–1.062) Ref. 1.08 (1.01–1.15) 1.07 (1.00–1.45) 1.23 (1.08–1.39) 1.60 (1.20–2.14)	1.026 (0.999–1.054) Ref. 1.06 (1.00–1.14) 1.06 (1.00–1.13) 1.18 (0.99–1.39) 1.48 (0.99–2.23)

CPA, collagen proportionate area; HR, hazard ratios.

Higher CPA was also associated with an increased all-cause mortality with a HR of 1.04 (1.01–1.07). Results attenuated somewhat but remained largely consistent when adjusting for covariates. When scaling HRs based on the median CPA values observed in different groups, the HRs were somewhat smaller in all models compared to those obtained from fibrosis stage analyses.

Likelihood ratio tests comparing models with and without the interaction between alcohol and fibrosis stage (both as measured by pathologist and by CPA) did not improve model fit (*P* = 0.78 for fibrosis stage and *P* = 0.80 for CPA). Likewise, AIC values were lower in the models excluding the interaction terms, both for fibrosis stage (971 vs 977) and for CPA (969 vs 971). For this reason, we retained the simpler model without the interaction term between fibrosis stage and alcohol for subsequent analyses.

During a mean follow-up of 14.1 (IQR: 6.1–19.4) years, the combined liver outcome was observed in 49 patients, and death from non–liver-related outcomes in 71 patients. Among fibrosis groups, 8 (18 %) individuals with F0, 10 (24 %) individuals with F1, 7 (30%) individuals with F2, 19 (45 %) individuals with F3, and 5 (36%) individuals with F4 developed a liver outcome. The incidence rates were 1.24, 1.64, 2.50, 3.78, and 4.28 events per 100 person-years, respectively.

In the unadjusted model, there was a monotonic relationship between fibrosis stage and liver outcomes. Compared with F0, the association was significant for F3 with an HR of 3.25 (95% CI: 1.41–7.45) and for F4 with an HR of 4.02 (1.29–12.52). When adjusting for alcohol and age, HRs were generally stable, and the associations for F3 and F4 remained statistically significant. An increase in CPA was associated with an HR of 1.03 for liver-related events in both unadjusted and fully adjusted models. While similar in magnitude to the HR observed for all-cause mortality, this association was not statistically significant (Table 3).

**Table 3:** Hazard Ratios (HR) for Fibrosis Assessed by Pathologists or by Collagen Proportionate Area (CPA) and Liver Events. Model 1: Unadjusted. Model 2: Adjusted for Alcohol. Model 3: Further Adjusted for Age, Hypertension, and Type 2 Diabetes Mellitus. Scaled CPA Estimates are Obtained by Applying the HR per % CPA Increase to the Difference in Mean CPA Values Between F1–F4 and F0.

	Variable	Model 1	Model 2	Model 3
		HR	HR	HR
Fibrosis stage	F0 F1 F2 F3 F4	Ref. 1.36 (0.54–3.44) 2.08 (0.75–5.76) 3.25 (1.41–7.45) 4.02 (1.29–12.52)	Ref. 1.38 (0.54–3.51) 2.26 (0.81–6.30) 3.18 (1.38–7.30) 3.86 (1.24–12.06)	Ref. 1.43 (0.55–3.71) 2.19 (0.77–6.24) 3.08 (1.32–7.16) 4.59 (1.39–15.17)
CPA Scaled CPA estimates	CPA (% increase) CPA 4.10 % (F0) CPA 6.52 % (F1) CPA 6.43 % (F2) CPA 10.42 % (F3) CPA 19.36 % (F4)	1.031 (0.989–1.074) Ref. 1.08 (0.97–1.20) 1.07 (0.96–1.19) 1.21 (0.96–1.54) 1.66 (1.06–2.60)	1.026 (0.984–1.071) Ref. 1.07 (0.96–1.18) 1.06 (0.95–1.17) 1.19 (0.94–1.50) 1.58 (1.01–2.46)	1.032 (0.989–1.077) Ref. 1.08 (0.97–1.20) 1.08 (0.98–1.19) 1.22 (0.93–1.60) 1.62 (0.85–3.10)

CPA, collagen proportionate area; HR, hazard ratios.

**Figure 4:**
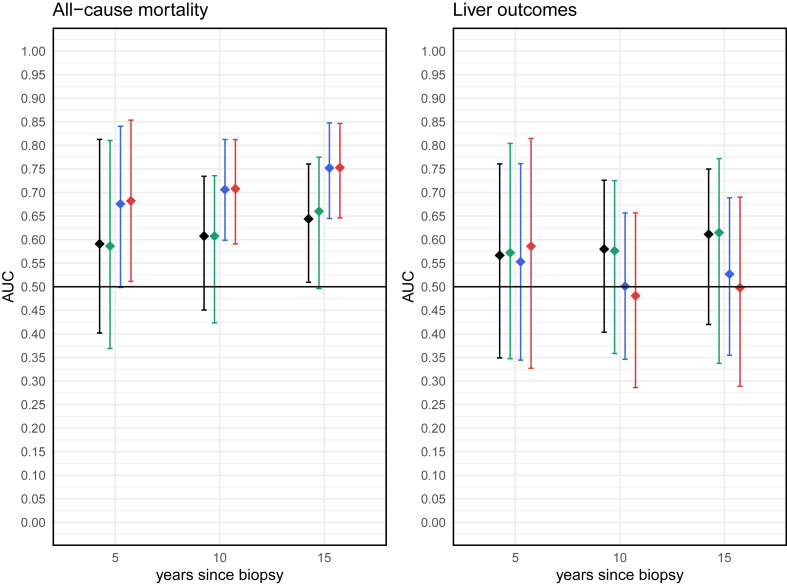
AUCs of various models and outcomes at different timepoints, internally validated by bootstrapping. **Black**: Fibrosis stage + alcohol. **Green:** CPA + alcohol. **Blue:** Fibrosis stage + alcohol + age + HT + T2DM. **Red:** CPA + alcohol + age + HT + T2DM. AUCs, area under the receiver operating characteristic curves; CPA, collagen proportionate area; HT, hypertension; T2DM, type 2 diabetes mellitus.

For the models of all-cause mortality including only alcohol, the AUC for fibrosis stage ranged between 0.59 (95% CI: 0.40–0.81) at 5 years and 0.64 (95% CI: 0.51–0.76) at 15 years, whereas the AUC for CPA ranged between 0.59 (95% CI: 0.37–0.81) at 5 years and 0.66 (95% CI: 0.50–0.78) at 15 years. When adjusting for covariates in these models, the AUC for fibrosis stage range between 0.68 (95% CI: 0.50–0.84) at 5 years and 0.75 (95% CI: 0.64–0.85) at 15 years, and the AUC for CPA ranged between 0.68 (95% CI: 0.51–0.85) at 5 years and 0.75 (95% CI: 0.65–0.85) at 15 years (Figure 4). The naïve AUC estimates were slightly higher values with narrower confidence intervals (Supplementary 4). For liver outcomes, in models including only alcohol, the AUC for fibrosis stage ranged between 0.57 (95% CI: 0.35–0.76) at 5 years and 0.61 (95% CI: 0.42–0.76) at 15 years. The AUC for CPA ranged between 0.57 (95% CI: 0.35–0.80) at 5 years and 0.61 (95% CI: 0.34–0.77) at 15 years. Including covariates in the models worsened AUCs at all follow-up times, with the lowest AUC being 0.48 (95% CI: 0.29–0.66) for CPA at 10 years (Figure 4). IPA values for all-cause mortality were positive in all-cause mortality, comparable between fibrosis stage and CPA, and increased over time throughout the study period (Supplementary 5). For liver-related outcomes, the IPAs values were consistently poor (mostly negative) and further worsened when covariates were included in the model. Calibration plots showed fairly good prediction of absolute risks in all models, with slightly better calibration for CPA compared with fibrosis stage in models without covariate adjustment (Supplementary 7). For Liver outcomes, calibration was poor with a tendency to underestimate absolute risks across all models (Supplementary 8).

## DISCUSSION

In this study of patients with biopsy-confirmed SLD, we observed a significant correlation between pathologist-assessed fibrosis stage and CPA, particularly among those with advanced fibrosis.

Both fibrosis stage and CPA were associated with all-cause mortality over long-term follow-up. However, only individuals with cirrhosis (F4) had significantly elevated mortality risk compared with those without fibrosis. CPA was independently associated with mortality, and its predictive value remained after adjustment for alcohol consumption but became nonsignificant when further adjusted for age. Compared to the association between fibrosis stage and mortality, the association for CPA was weaker in scaled estimates.

For liver-related outcomes, risk increased with higher fibrosis stages, especially F3 and F4, and these associations remained significant after full adjustment. CPA showed a similar trend, though without reaching statistical significance for liver-specific events. Scaled estimates behaved similarly to those for all-cause mortality.

When assessing model performance for predicting all-cause mortality, both fibrosis stage and CPA demonstrated comparable discriminative ability for all-cause mortality and liver-related events. Discriminative ability was acceptable for both outcomes but both fibrosis stage and CPA tended to underestimate the absolute risk of liver events. The comparable performance of CPA and fibrosis stage likely reflects their biological overlap and the methodological constraints of histopathologic evaluation, particularly in distinguishing early disease.

Both the degree of correlation and the pattern of higher correlation in higher fibrosis stages are comparable with previous studies.^27^^,^^28^ It should be noted that poor correlation at lower fibrosis stages may not necessarily reflect poor performance. CPA represents a quantitative measurement of fibrosis extent, whereas fibrosis staging reflects both the location and extent of fibrosis. The fibrosis score is derived from the extent of zone 3 perisinusoidal fibrosis (F1a-c) with possible additional portal/periportal fibrosis (F2), and architectural remodeling (F3 and F4 for bridging fibrosis and cirrhosis, respectively).^29^

Discriminative ability for all-cause mortality and liver-related mortality was somewhat smaller than in previous studies. This may partly be explained by the relatively small sample size and the lack of consistent internal validation in prior studies, with prior estimates being partly biased due to overfitting. However, even the naïve estimates in our study were lower than those previously observed.^27^^,^^28^^,^^30^ To our knowledge, predictive performance of CPA and fibrosis stages apart from discrimination has not been performed in previous studies.

A key limitation of this study is the smaller-than-expected cohort size, with only 166 individuals included versus the anticipated 800. The primary reason was the difficulty in obtaining the requested materials from pathology departments. The relatively large number of retrieved samples later excluded due to diagnoses other than SLD may also suggest suboptimal sampling. However, some degree of material exclusion was expected because of small biopsies or occasional misclassification in the original pathology assessments.

This limited sample size results in wider confidence intervals and reduces statistical power, making it more difficult to detect subtle associations or produce precise estimates. The relatively small population also restricts the robustness of predictive modeling, especially for liver-related outcomes; therefore, these results should be interpreted with care. Furthermore, it was not possible to assess the indication for liver biopsy. As such, mortality for lower fibrosis stages may be overestimated if there was some other indication for possible disease present, not captured by the exclusion criteria. In fact, the increased mortality by increasing fibrosis score in the present study was less than that observed in prior studies.^31^ Due to the use of a general patient registry, it was not possible to assess biochemical or clinical variables such as liver function tests, which may limit the interpretability of the risk prediction models. However, regarding the comparison of fibrosis staging and CPA, it is unlikely that they should relate to these in unequal ways. Finally, the biopsy material predates recent changes in MASLD nomenclature and the widespread adoption of noninvasive assessments. Nonetheless, fibrosis stage and CPA remain core histologic markers of liver disease severity, and the extended follow-up available in this cohort offers clinically relevant long-term outcome data.

Of note, we observed a relatively high number of liver-related events in patients with no or minimal fibrosis (F0–F1). This finding may reflect selection bias or possible misdiagnosis of outcomes among individuals initially suspected of having liver disease. However, since all biopsies were re-evaluated using newly sectioned slides by the same pathological criteria, any misclassification of fibrosis stage is likely to be nondifferential and limited.

On the other hand, the study's strengths include extensive long-term follow-up and highly accurate mortality data derived from national registries, ensuring reliable outcome ascertainment. Prior studies have not consistently applied internal validation.^27^^,^^30^ Estimating model performance on the whole fitted dataset may lead to bias arising from overfitting. The use of internal validation with bootstrapping enhances the credibility of the predictive performance estimates by mitigating overfitting.

Despite the limitations of this study, the results suggest that CPA is both closely correlated to fibrosis stage and performs comparably in long-term prediction. CPA showed comparable, but not superior prognostic value. These findings underscore the potential utility of CPA as a complementary measure to fibrosis staging in risk stratification of SLD. However, larger long-term studies with external validation are warranted to further validate these results and to explore whether integrating CPA into clinical and research practice could improve prognostication and patient management.

The increasing digitization of pathology workflows opens new avenues for standardized, quantitative fibrosis assessment. Although CPA did not outperform conventional staging, it may offer practical advantages, including reduced interobserver variability and continuous quantification, supporting its role as a complementary measure. Digital image analysis techniques, such as CPA, could help mitigate variability and provide continuous metrics that support risk stratification. As digital pathology infrastructure expands, particularly in countries like Denmark with centralized health data systems and structured pathology registries,^32^ the integration of automated fibrosis quantification into clinical workflows, as well as research settings, may become more feasible. This could enhance consistency in biopsy interpretation and facilitate large-scale studies using archival tissue.

## CREDIT AUTHORSHIP CONTRIBUTION STATEMENT

AB, LLG, and EDG conceived the study. LHV, PY and AB designed the methodology for data obtainment. MKJ acquired funding and provided the primary supervision. BMV and AB conducted the processing of materials. AB carried out data curation, analysis, figure creation, and wrote the initial manuscript draft. All authors reviewed and revised the manuscript before submission.

## Data Availability Statement

Data can be made available upon reasonable request. Access can be granted through collaborative agreements and directly through Statistics Denmark and the Danish Health Data Authorities. Please contact Professor Majken K Jensen (maje@sund.ku.dk) for further information.

## ETHICAL STATEMENT

The study was approved by the Regional Committee on Health Research Ethics (H-19078472). As this was a retrospective study, informed consent was not obtained.

## FUNDING

Funded by the Novo Nordisk Foundation (NNF17OC0027812).

## DECLARATION OF COMPETING INTEREST

MKJ has received a consultant fee from Pfizer on an unrelated liver project. LLG has received grants from Novo Nordisk, Alexion, Pfizer, Beckton Dickinson, Bayer, and Sobi International, and personal fees from AstraZeneca, Novo Nordisk, and Boehringer Ingelheim not related to this work. BMV and EDG are employees of and shareholders in Novo Nordisk A/S.
