# Supplementary material for: Collagen Proportionate Area and Histological Grading of Fibrosis as Predictors of Clinical Outcomes and Mortality in Patients with Steatotic Liver Disease
Source: J Clin Exp Hepatol. 2026 Jan 2;16(2):103464. doi: 10.1016/j.jceh.2025.103464 (PMC12860911; doi:10.1016/j.jceh.2025.103464)
Supplement: Multimedia component 1 [file mmc1.pdf]

Supplementary

S1a. Codes used to assess exclusion parameters

	Groups excluded	ICD-10	ICD-8	SNOMED-CT (in liver biopsy)
Viral Hepatitis	Hepatitis B, Hepatitis C	B(16-19)	700(0-9)	S0520(0-2), S05204, S05252, S05255
Other Causes for Chronic Liver Disease	Hemochromatosis, Wilsons disease, Alfa-1-antitrypsin deficiency, glycogen storage diseases, Budd-Chiari, Toxic liver disease, Autoimmune hepatitis, primary/secondary biliary cirrhosis, Congestive hepatopathy, HIV/AIDS, cholangitis, celiac disease, hepatic cancers.	B20, B(22-24), E740, E831A, E880B, E830B, I820, K71, K732, K744, K745, K75(1-4), K761, K830, K900	27109, 27119, 57191(0-2), 27329, 155(0-1)9, 15589, 19789, 57504, 26900, 27080, 27339, 45301, 5730(1-2), 7983	M49580, M49590, M49592, M49691, S10700, S11920, S63530, S63580, S6360(5-6), S63608, S63610, S63630, S97250, SYY610, SYY636<br><br>And any Code beginning with M8 or M9 and ending in 3, 6 or 9
Hepatocellular carcinoma		C220		M817*
Cirrhosis		K703, K704, K717, K74, K76	57109, 57190, 57191, 57192	M4950(0-1), M4950(3-6), M49610, M49514, M49520, M49524, M4952(6-8), M49530, M49570, M49660, M49690

S1b. codes used to assess liver biopsies for steatosis and fibrosis

	Snomedcodes
Steatosis	M50085 or M50086
Steatohepatitis	M45400
Fibrosis codes	M45000, M49000, M49001, M49002, M49005, M49230, M49232, M49620

S2. Definition of excessive alcohol consumption, diabetes, hypertension and dyslipidemia

		ATC codes used for prescriptions	ICD-10 codes	ICD-8 codes
Diabetes	Two purchases of antidiabetic drugs within 180 days, and at least one purchase of a non-insulin within this timeframe	A10A (insulins)<br>A10B (non-insulins)		
Hypertension	Two purchases of antihypertensives within 180 days	C02A, C02DB, C02DD, C03, C07, C08, C09A, C09C, G04CA		
Dyslipidemia	Two purchases of lipid lowering drugs within 180 days	C10		
Excessive alcohol consumption	Any registration of following codes		E224, G312, G721, I426, K292, K70, K852, K860, T500A, X65, Z502, Z714, Z721, F(10-19)	291, 303, 57109, 57110, 57710, 979, 304(0-9)9

S3. Components and icd10 codes of the compound liver outcome

Category	ICD-10 codes
Decompensated liver events	K766, K767, R17, R18, I850, I865A, K704, K720, G943, K72
Oesophagal varices without bleeding	I864, I85, I982
Liver Cancer	C22
Liver death (base on ICD code registered as underlying cause of death)	K70-77 or C22

S4. Naïve AUCs of various models and outcomes at different timepoints.
Black: Fibrosis stage + alcohol.
Green: CPA (collagen proportionate area) + alcohol.
Blue: Fibrosis stage + alcohol + age + HT (hypertension) + T2DM (type 2 diabetes mellitus).
Red: CPA + alcohol + age+ HT + T2DM.

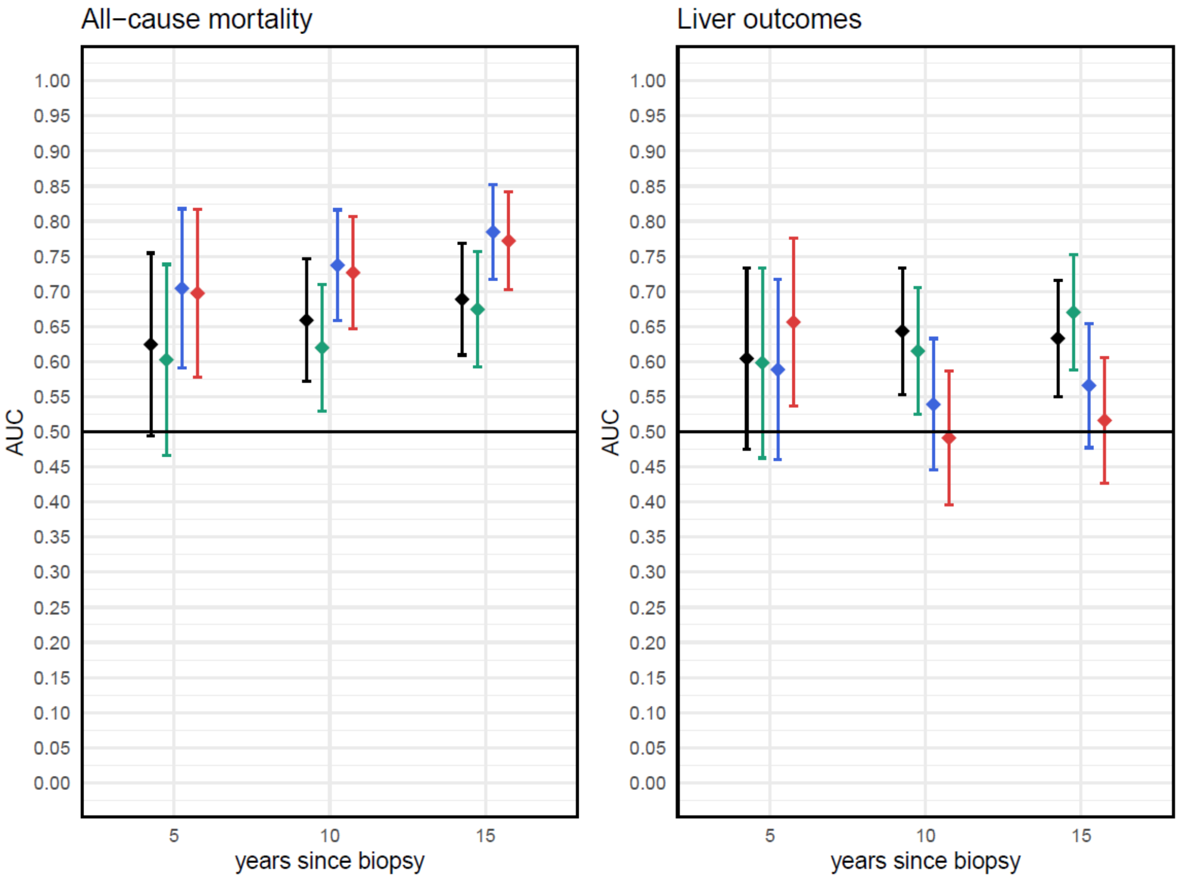

S5. Index of Prediction Accuracy (IPA) of various models and outcomes at different timepoints internally validated by bootstrapping.
Black: Fibrosis stage + alcohol.
Green: CPA (collagen proportionate area) + alcohol.
Blue: Fibrosis stage + alcohol + age + HT (hypertension) + T2DM (type 2 diabetes mellitus).
Red: CPA + alcohol + age+ HT + T2DM.

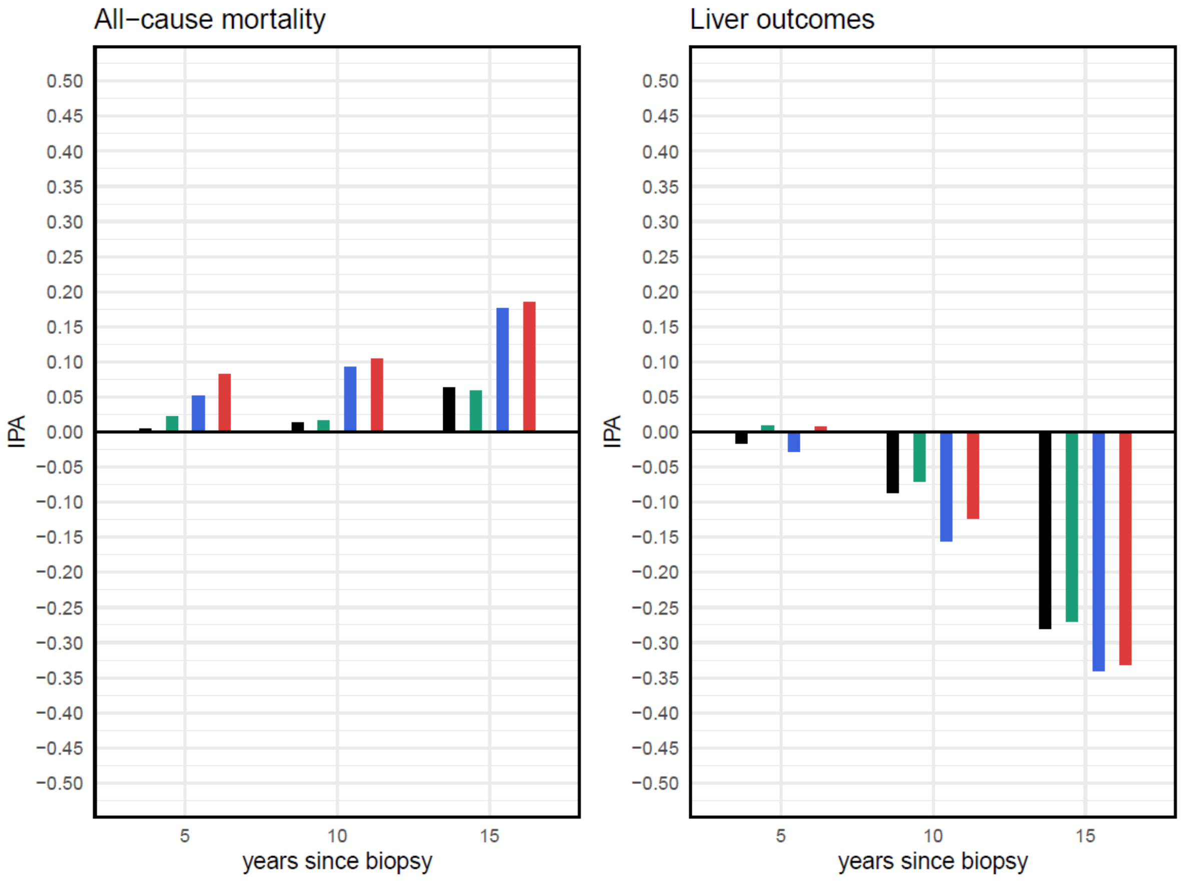

S6. Naïve index of Prediction Accuracy (IPA) of various models and outcomes at different timepoints.
Black: Fibrosis stage + alcohol.
Green: CPA (collagen proportionate area) + alcohol.
Blue: Fibrosis stage + alcohol + age + HT (hypertension) + T2DM (type 2 diabetes mellitus).
Red: CPA + alcohol + age+ HT + T2DM.

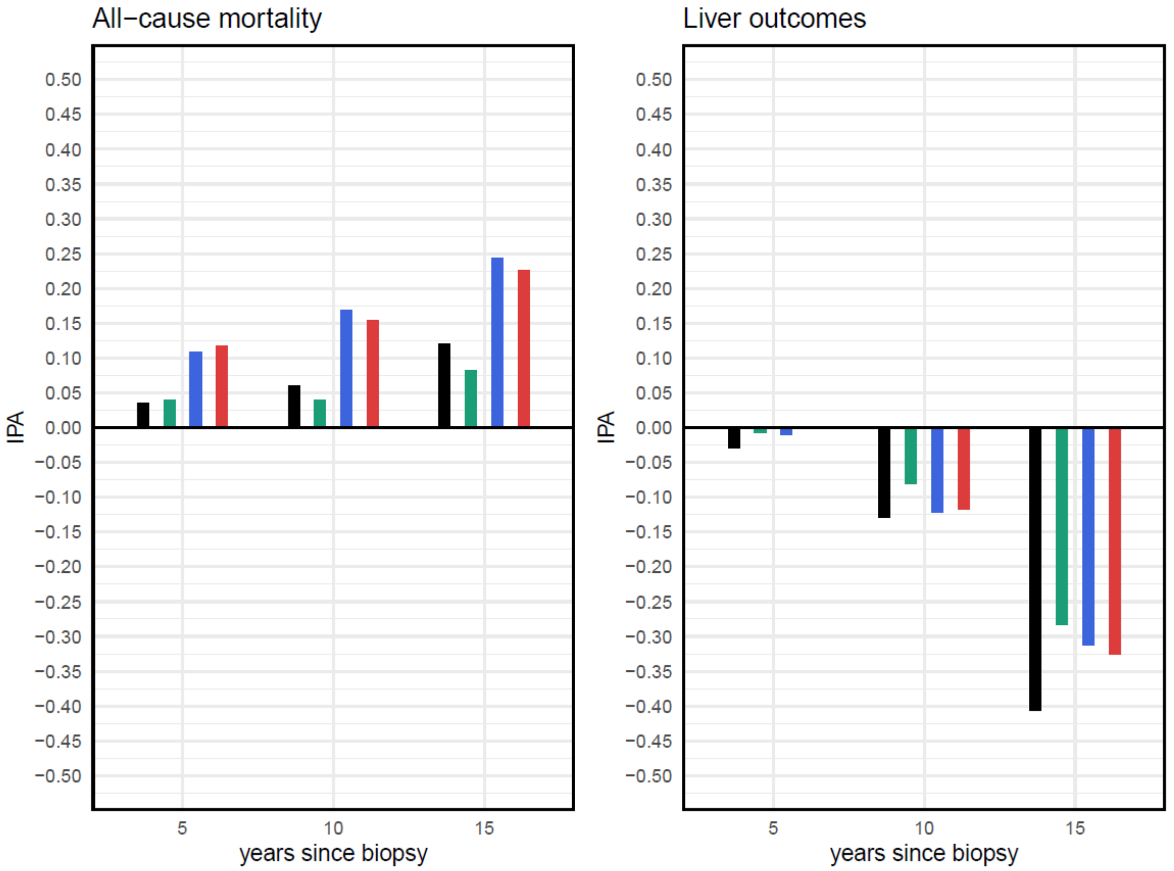

S7. Bootstrapped and naïve calibration plots for various models and death at 5, 10, and 15 years.
Black: Fibrosis stage + alcohol.
Green: CPA (collagen proportionate area) + alcohol.
Blue: Fibrosis stage + alcohol + age + HT (hypertension) + T2DM (type 2 diabetes mellitus).
Red: CPA + alcohol + age+ HT + T2DM.

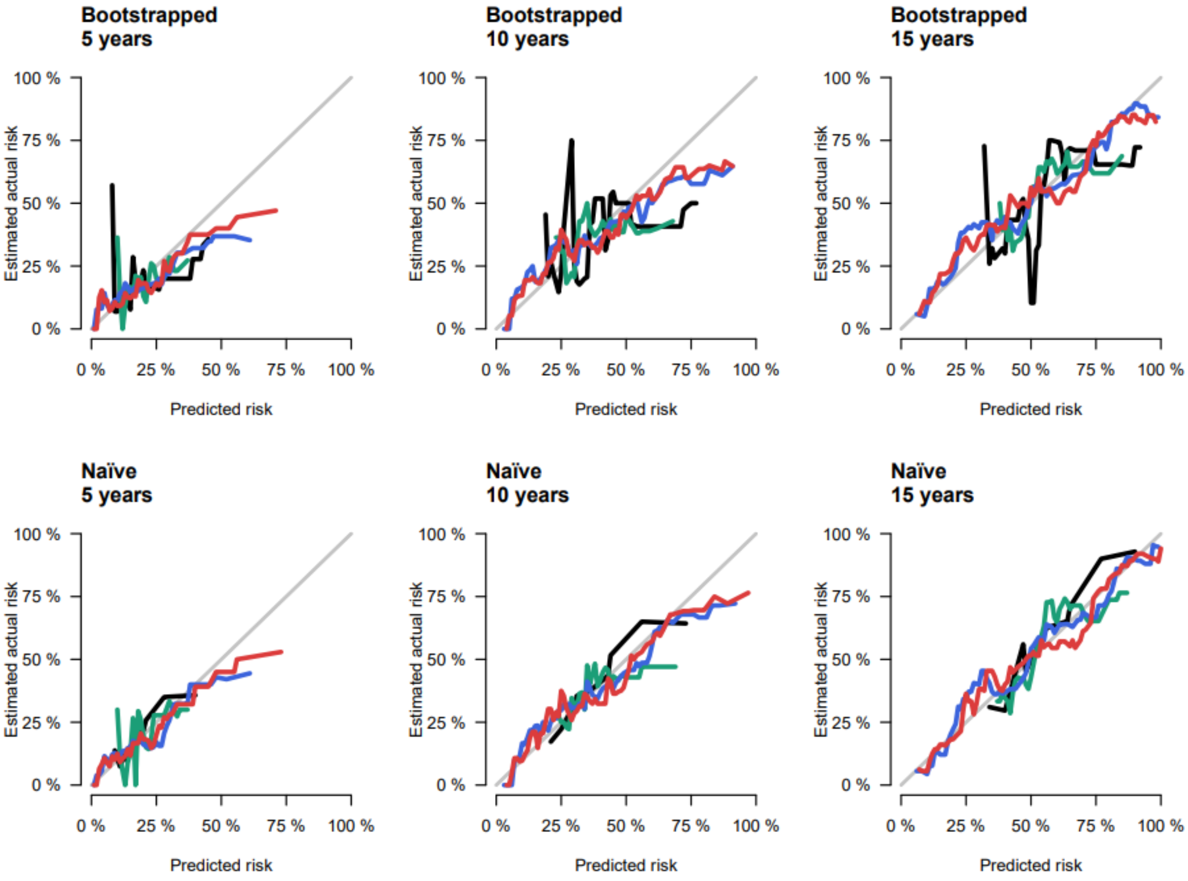

S8. Bootstrapped and naïve calibration plots for various models and liver outcomes at 5, 10, and 15 years.

Black: Fibrosis stage + alcohol.

Green: CPA (collagen proportionate area) + alcohol.

Blue: Fibrosis stage + alcohol + age + HT (hypertension) + T2DM (type 2 diabetes mellitus).

Red: CPA + alcohol + age+ HT + T2DM.

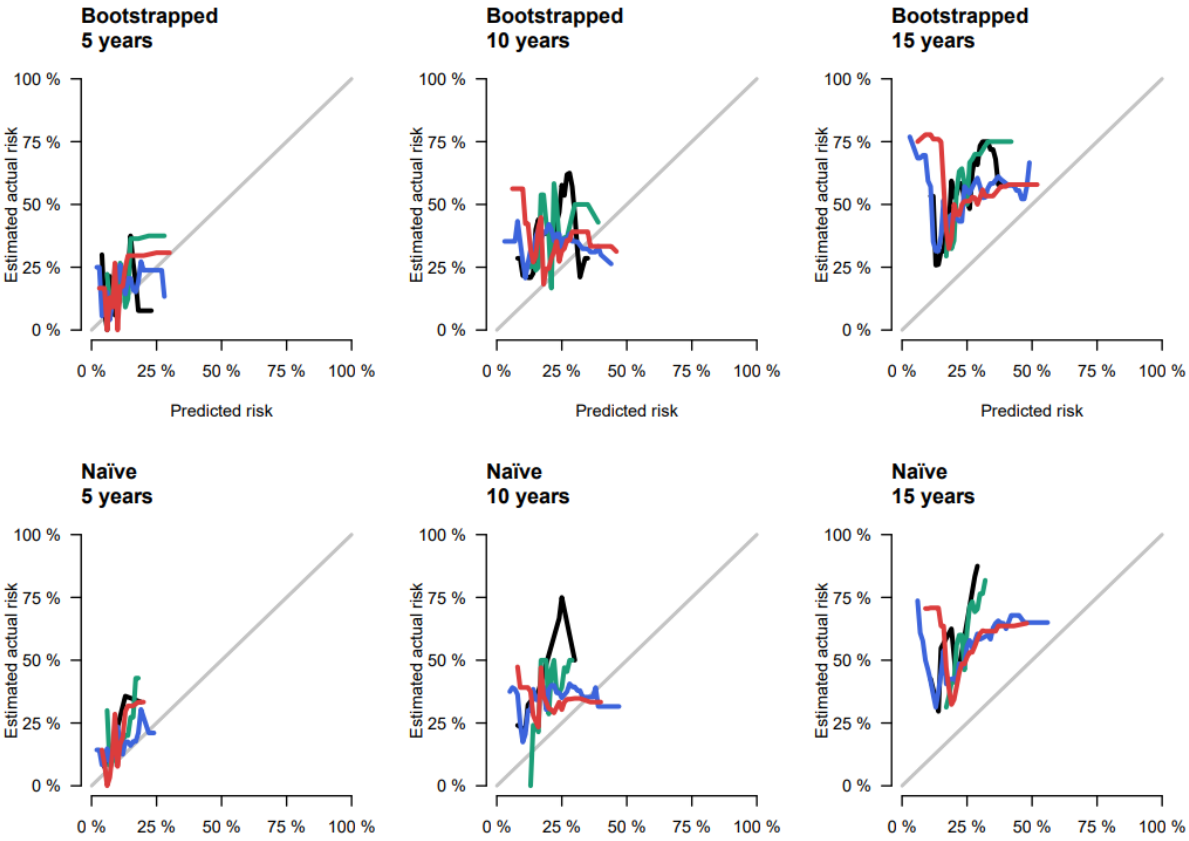

S9. Comparison of observed variables and standardized mean differences (SMD) in patients whose biopsies were retrieved vs not retrieved.

Variable	Not received biopsies	Received biopsies	SMD
Total, n	238	546	
Male, n (%)	123 (52%)	220 (40%)	0.23
Diabetes, n (%)	26 (11%)	68 (12%)	0.05
Hypertension, n (%)	111 (47%)	273 (50%)	0.07
Dyslipidemia, n (%)	13 (5%)	63 (12%)	0.22
Alcohol overconsumption, n (%)	82 (34%)	179 (33%)	0.04
Age, mean (sd)	52.6 (12.7)	53.7 (12.8)	0.09
